# Clinical application of a digital semi-rigid bridge space maintainer fabricated from polyetheretherketone for premature loss of primary molars

**DOI:** 10.1186/s12903-023-03570-2

**Published:** 2023-11-29

**Authors:** Qi Wang, Zhenzhen Zhang, Sheng Zhong, Jiajia Liu, Ying Hu, Ziling Zhou, Caidi Zhang, Shizhu Bai, Li’an Wu

**Affiliations:** 1 State Key Laboratory of Oral & Maxillofacial Reconstruction and Regeneration, National Clinical Research Center for Oral Diseases, Shaanxi Clinical Research Center for Oral Diseases, Department of Pediatric Dentistry, School of Stomatology, The Fourth Military Medical University, Xi’an, Shaanxi China; 2 State Key Laboratory of Oral & Maxillofacial Reconstruction and Regeneration, National Clinical Research Center for Oral Diseases, Shaanxi Key Laboratory of Stomatology, Department of Prosthodontics, School of Stomatology, The Fourth Military Medical University, Xi’an, Shaanxi China; 3 State Key Laboratory of Oral & Maxillofacial Reconstruction and Regeneration, National Clinical Research Center for Oral Diseases, Shaanxi Key Laboratory of Stomatology, Digital Dentistry Center, School of Stomatology, The Fourth Military Medical University, Xi’an, Shaanxi China; 4 Digital Dentistry Center, School of Stomatology, The Fourth Military Medical University, No. 145, Changle West Road, Xincheng District, Xi’an, Shaanxi China; 5 Department of Pediatric Dentistry, School of Stomatology, The Fourth Military Medical University, No. 145, Changle West Road, Xincheng District, Xi’an, Shaanxi China

**Keywords:** Polyetheretherketone, Semi-fixed bridge, Space maintenance, Computer-aided design, Premature loss of primary molar

## Abstract

**Background:**

Premature loss of primary molars can be treated with a band loop space maintainer (SM). However, fabricating a conventional band loop SM requires multiple clinical and laboratory procedures, which can potentially affect the accuracy of the SM. Moreover, the conventional SM is unable to fully restore masticatory function and maintain the vertical dimension of the edentulous space. In this current study, a fully digital workflow to fabricate a semi-rigid bridge SM made from polyetheretherketone (PEEK) has been described and evaluated for its clinical effectiveness.

**Methods:**

A total of 15 children (eight males and seven females) between the ages of 4–8 years, who experienced the premature loss of a single primary molar, were included in this study. Digital impressions were taken using the CEREC CAD/CAM chair system and imported into CAD software to design the semi-rigid bridge SM, which was fabricated using PEEK block as the maintainer material. The digital SM was tried-in and bonded to the abutment with resin cement. The edentulous space was measured immediately after bonding (T0) and 1 month (T1), 3 months (T2), and 6 months (T3) after treatment. The periodontal condition and mobility of the SM and abutment were also examined.

**Results:**

The use of digital impressions resulted in a decreased occurrence of the pharyngeal reflex. The digital semi-rigid bridge SM, fabricated with PEEK, was both convenient and aesthetically pleasing, and successfully restored the anatomy and masticatory function of the missing primary molar. None of the 15 semi-rigid bridge SMs or abutments became loose or fell off during the study, and only one child presented with gingivitis. Furthermore, the difference in the edentulous space at T0, T1, T2, and T3 was not statistically significant (all P > 0.05).

**Conclusions:**

The digital semi-rigid bridge SM fabricated with PEEK was clinically effective in maintaining the missing space and had advantages over the traditional band/crown loop SM.

## Background

Premature loss of primary teeth can lead to reduced masticatory function and edentulous spaces, affecting permanent teeth’ normal eruption and increasing the risk of malocclusion [1]. Therefore, managing the space after the early loss of primary molars is crucial. Band/crown loop space maintainer (SM) is commonly used for unilateral premature loss of a single primary molar. However, its fabrication involves alginate elastic impression material, which can induce discomforts like breathing difficulty and severe gag reflex, making it challenging for pediatric patients to cooperate [2]. Moreover, complications such as band breakage, debonding, desoldering, and loop embedding into the gingival may occur [3]. Notably, the metal material of band loop SM falls short in aesthetics and fails to restore masticatory function [4, 5].

In recent years, intraoral digital scanners and computer-aided design and manufacturing (CAD/CAM) have become increasingly popular in dentistry. Polyetheretherketone (PEEK) is an exceptional engineering plastic with desirable mechanical performance, high temperature and corrosion resistance, and excellent biocompatibility [6]. PEEK has been used to fabricate fixed and removable partial dentures [7, 8]. A pilot study has indicated that PEEK is a suitable material for the production of SM [9]. Moreover, PEEK exhibits excellent cutting performance and can be easily shaped using CAD/CAM technology.

This study aimed to introduce a fully digital workflow for fabricating semi-rigid bridge SMs using CAD/CAM technology and to evaluate their clinical effectiveness.

## Methods

### Patient selection

A total of 15 children who attended the Pediatric Dentistry Department at the Third Affiliated Hospital of the Air Force Military Medical University between April and June 2022 were included in the study. Inclusion criteria were as follows: (1) age range of 4–8 years with the ability to cooperate with clinical treatment; (2) premature loss of a single first primary molar with earlier development of permanent successor than Nolla7 and its surface covered with bone; (3) no mobility or root resorption, or if present, the root resorption was less than 1/3 of the second primary molar and primary canine (abutment tooth); (4) no caries of the abutment tooth or the abutment tooth underwent caries/pulp therapies; (5) informed consent obtained from both the children and their parents and willingness to undergo regular follow-up. Exclusion criteria included: (1) poor compliance and inability to maintain good oral hygiene by the children and their parents; (2) root resorption of the abutment tooth exceeding 1/3. The new technology of the digital SM was approved by the School of Stomatology, the Fourth Military Medical University, under registration number IRB-REV-2019023.

### Digital design and fabrication of semi-rigid bridge SM

**Fig. 1:**
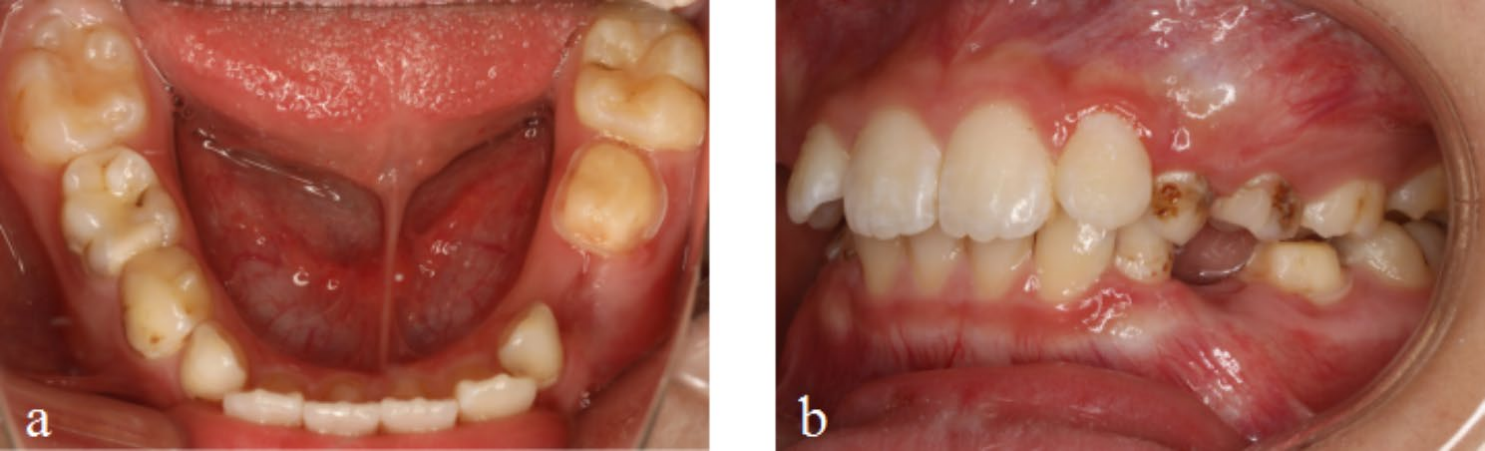
Preoperative view (1a mandibular occlusal view; 1b lateral view)

Example patient: An 8-year-old girl presented with premature loss of tooth 74 due to periapical periodontitis. Tooth 75 underwent root canal therapy and standardized tooth preparation for a composite resin crown, including 1.5 mm of occlusal surface preparation and 1.0 mm of buccal, lingual, and proximal surface preparation, with a shoulder of 1.0 mm (Fig. 1). The treatment plan was discussed with the patient’s parents and proceeded with a semi-rigid bridge SM.

Digital data acquisition of the primary teeth involved scanning the plaster cast of the dentition without teeth or dentition defects using the CEREC CAD/CAM system (Sinold, Germany). The resulting files were saved in STL format and imported into CAD software (Exocad DentalCAD, Exocad GmbH Company, Germany). Each scanned primary tooth model was then copied one by one based on its tooth position, and the copied tooth data were trimmed and annotated for the mesial, distal, buccal, and lingual aspects to create separate tooth morphology models.

#### Digital model:

**Fig. 2:**
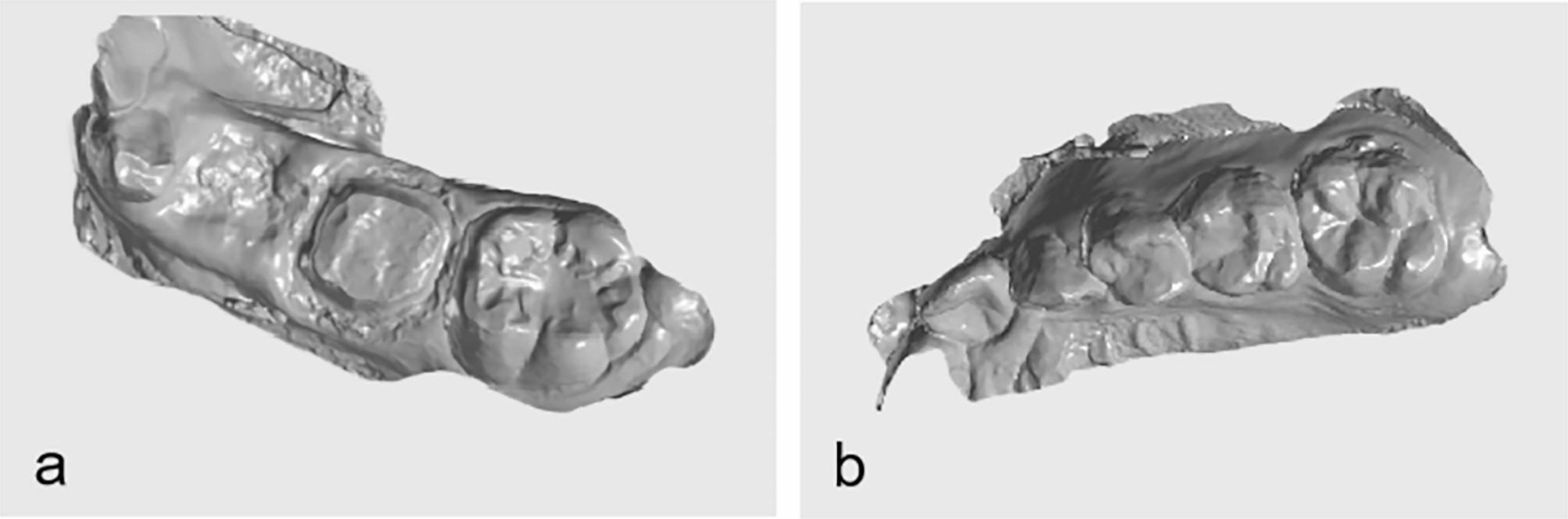
The digital model (2a mandibular model; 2b maxillary model)

The CEREC CAD/CAM system was used to obtain digital maxillary and mandibular models of the side with premature loss and occlusion relationship, which were saved in STL format (Fig. 2).

**Design of semi-rigid bridge SM**: The semi-rigid bridge SM consisted of three parts: the retainer, pontic, and connector. The files in STL format were imported into the CAD software, and the path of insertion was determined.

**Fig. 3:**
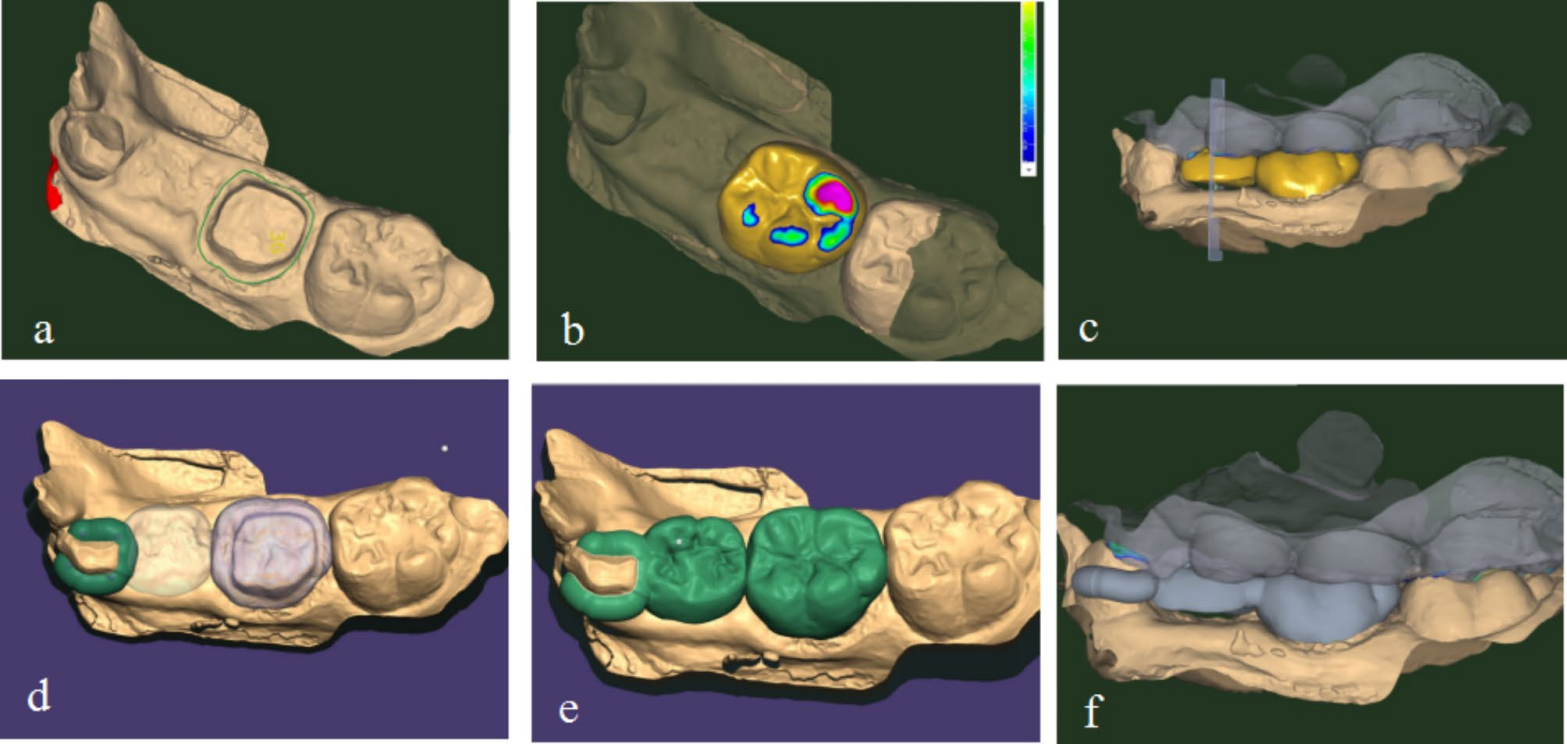
Digital design of semi-rigid bridge SM (full crown) (3a determined edge line; 3b full crown design; 3c pontic design; 3d non-rigid connection retainer design; 3e connector design; 3f check the occlusal relationship

Retainer design: The retainer had two parts. One end was a rigid connection, and the other end was a non-rigid connection. The retainer for the rigid connection was designed as a full crown or band based on the defect range of the abutment teeth. In this case, the retainer of the rigid connection was designed as a full crown. The placement of the edge line was determined by considering the position of the abutment tooth’s shoulder, pre-aligned tooth morphology from the database, and available bonding space. The occlusion, adjacent relationship, and other variables were also designed. Finally, the occlusal relationship was verified to avoid excessive occlusal contacts (Fig. 3a and b). The non-rigid connection retainer was designed as a two-arm clasp with a thickness of 1.5 mm and a width of 2.5 mm, avoiding the occlusal contact area (Fig. 3d).

Pontic design: In order to establish the appropriate orders within the software, the primary tooth data obtained by scanning the plaster cast were utilized to select the anatomical missing tooth that corresponds to the losing tooth position. Within the design module of the software, the pontic design should be personalized based on the morphology of the primary tooth and the occlusal relationship. To minimize the burden on the abutment teeth, it is recommended to appropriately reduce the buccolingual width of the pontic and the inclination of the cusp. Additionally, the pontic gingival types should be designed to be suspended with a 2–3 mm gap to the gingiva in the area of the missing tooth to facilitate food passage without accumulation (Fig. 3c).

Connector design: The connector should be positioned in the middle third of the partial occlusal surface, with a cross-sectional area of approximately 4 mm^2^, to create a normal embrasure and interproximal space (Fig. 3e). After designing all components, it is important to verify the occlusal relationship (Fig. 3f).

**Fig. 4:**
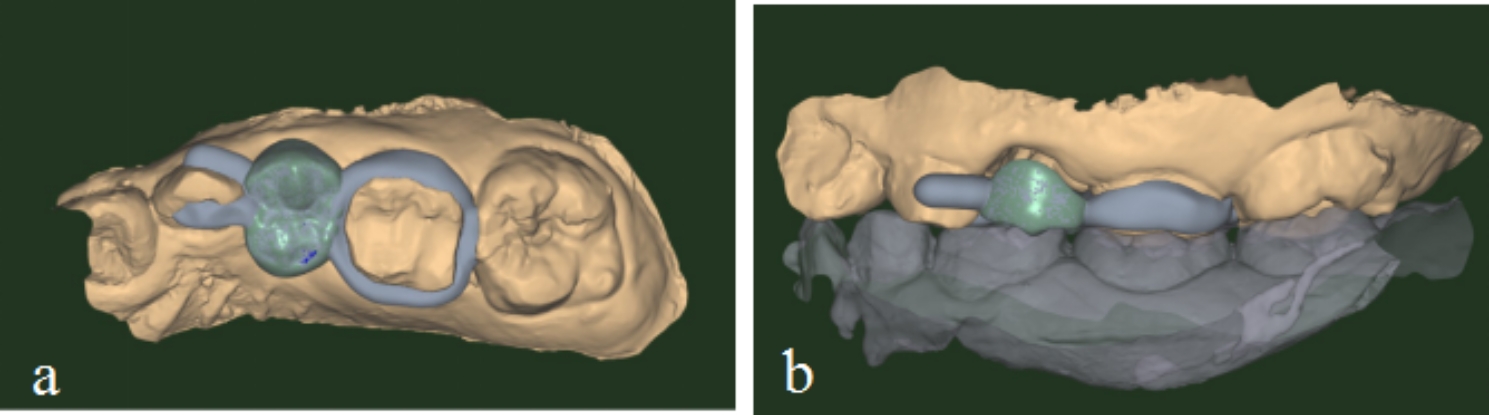
Digital design of semi-rigid bridge SM (band)

When the abutment tooth has no defects, the retainer can be designed as a band. The band’s form should be designed according to the appearance of the abutment and avoid any occlusal contact spots. The thickness should be designed as 1.5 mm, and the width should be designed according to the crown length of the abutment, stopping at a position 1 mm above the gingival margin. Other variables, such as abutment undercut depth of the band and adjacent relationships, should also be designed. The design of other components should be the same as described above (Fig. 4).

#### Fabrication of semi-rigid bridge SM:

**Fig. 5:**
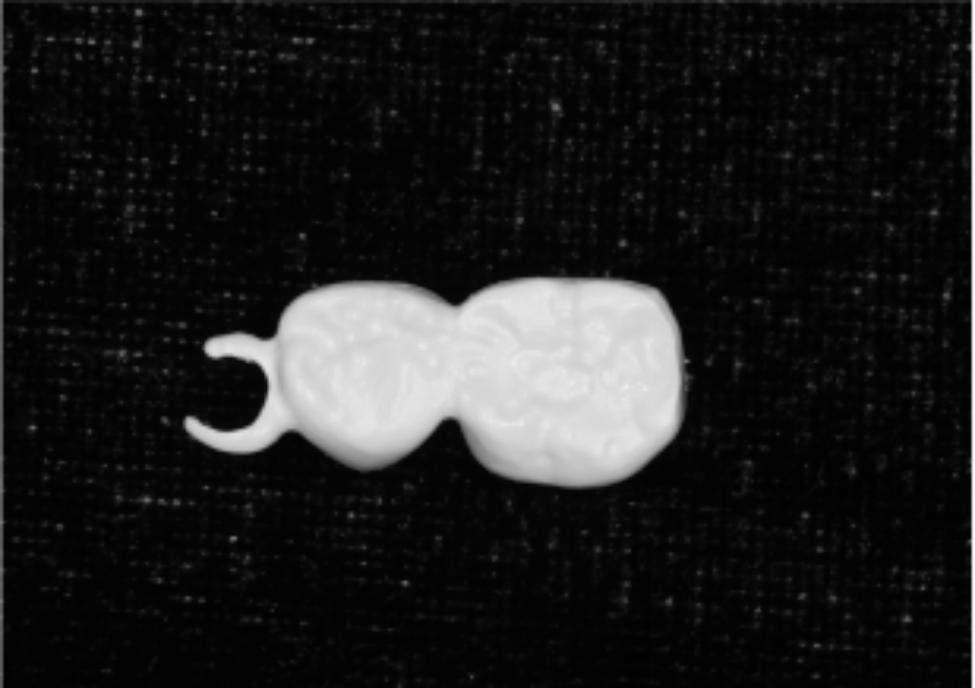
PEEK digital semi-rigid bridge SM

The designed data files of the digital semi-rigid bridge SM were imported into the milling equipment (Ceramiall Matik, Amann Girrbach Company, Germany). The SM should be cut and formed using PEEK (PEEK disc, Sino-Dentex Co., Ltd., China) (Fig. 5).

### Semi-rigid bridge SM try-in and bonding

**Fig. 6:**
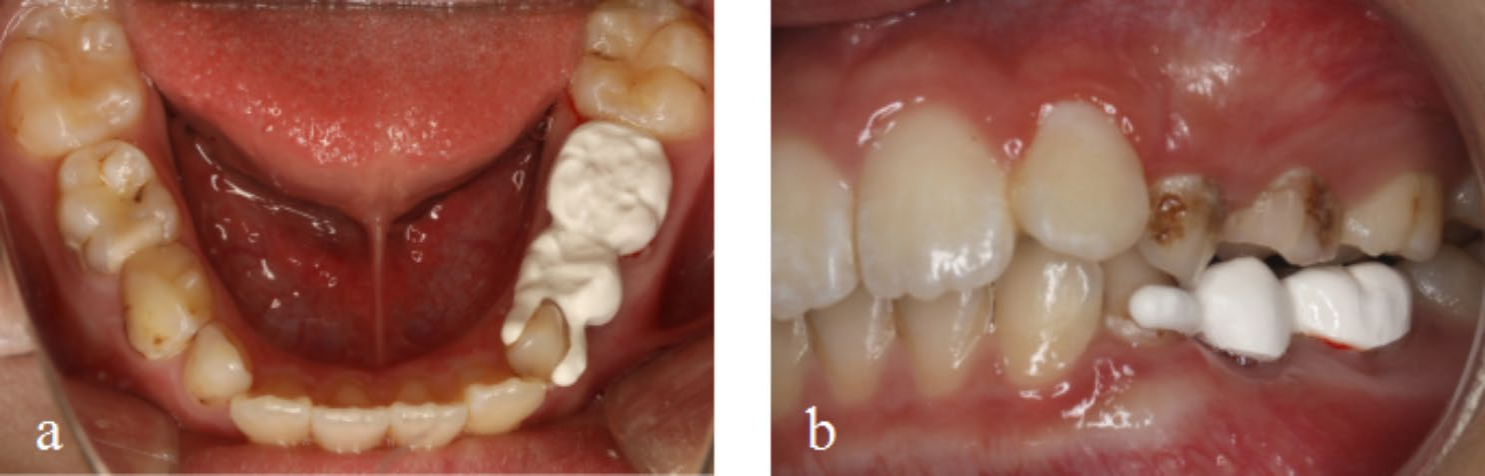
PEEK semi-rigid bridge SM view (6a mandibular occlusal view; 6b lateral view)

Tried in the semi-rigid bridge SM and checked the marginal adaptation, retention, occlusion, and the distance between the pontic gingival surface and the gingiva. If any adjustment was necessary during the try-in, we modified the bridge and then polished it again. Finally, the semi-fixed bridge SM was bonded with resin cement (Clearfil Sa Luting Dual Cure Dental Adhesive System; Kuraray Noritake Dental Inc., Okayama, Japan) under a rubber dam (Kerr Optidam; 3-Dimensional Rubber-Dam System, USA) isolation (Fig. 6).

The patient and their parents were duly informed of the critical significance of maintaining proper oral hygiene and adhering to scheduled follow-up appointments. Furthermore, they were counseled on the implementation of the Bath method and encouraged to incorporate dental floss or water floss for effective removal of food debris within the diastemata. The mesiodistal width of the edentulous space was measured immediately after bonding the SM (T0) and then at 1 (T1), 3 (T2), and 6 months (T3) post-treatment. The periodontal condition and the mobility degree of the SM and abutment were also examined. Additionally, the children and parents satisfaction with the aesthetics and masticatory function of the SMs were survied though oral questioning.

### Statistical analysis

Statistical analysis was performed using Statistica software for Windows (SPSS 20.0, IBM, USA). Normally distributed measurement data were represented with ${\bar {x}}$±S. The SNK-q test was used to analyze intragroup comparisons between observation times T0, T1, T2, and T3 for parametric data. P < 0.05 was considered statistically significant.

## Results

The design and fabrication of the digital semi-rigid bridge SM required approximately 1.5 h and could be accomplished on the same day as the treatment. Fifteen digitally fabricated semi-rigid bridge SMs fit seamlessly during the try-in, demonstrating good retention, marginal adaptation and occlusion relationship. The PEEK semi-rigid bridge SMs delivered excellent aesthetic performance, effectively replicating the morphology and masticatory function of prematurely lost primary teeth. Both the children and their parents expressed satisfaction with the aesthetics and function of the SMs. At the T1, T2, and T3 follow-ups, all 15 semi-rigid bridge SMs remained stable, with no observed breakage or dislodgement, and the abutment teeth displayed no abnormal mobility or caries. Only one child exhibited poor oral hygiene at the T1 follow-up, with considerable debris packing around the pontic area and grossly inflamed gingiva that appeared bright red in the edentulous area. The edentulous space did not demonstrate any significant difference between observation times (Table 1).

**Table 1:** Edentulous space in different tooth positions (${\bar {x}}$ ±S), mm

Tooth position	T0	T1	T2	T3	F	P
54 (3 cases)	7.33 ± 0.64	7.2 ± 0.49	7.53 ± 0.78	7.23 ± 0.71	0.300	0.825
64 (4 cases)	7.48 ± 0.07	7.4 ± 0.07	7.43 ± 0.28	7.4 ± 0.14	0.037	0.990
74 (4 cases)	9.05 ± 0.07	8.95 ± 0.28	9.1 ± 0.07	8.98 ± 0.41	0.318	0.812
84 (4 cases)	8.75 ± 0.71	8.63 ± 0.71	8.8 ± 0.57	8.6 ± 0.49	0.204	0.892

## Discussion

Primary teeth play a critical role in children’s growth and development, guiding the eruption of permanent teeth, promoting maxillofacial development, and maintaining arch length and normal occlusion. Premature loss of primary teeth due to caries or trauma not only affects masticatory function but can also reduce the arch length and increase the risk of malocclusion [10, 11]. Therefore, it is essential to install a semi-rigid bridge after primary tooth extraction until inherited permanent teeth erupt to prevent adjacent tooth inclination and antagonist tooth extrusion.

Currently, the band/crown loop SM is commonly used to maintain the space for unilateral premature loss of a single primary molar. However, the fabrication process of the traditional SM involves multiple steps, including selecting and trying on the band, making impressions, pouring and mounting casts, trimming dies, and bending the loop. The accuracy of the SM depends on the impression accuracy and the expertise of the technician, with the potential for human error in each step that may reduce accuracy or lead to SM failure. Additionally, there is no suitable band for the first primary molar, and preparing healthy primary teeth for the crown loop SM may be necessary when using the first primary molar as an abutment tooth. Furthermore, the traditional SM cannot fully restore the masticatory function or maintain the vertical space [5].

A technique for designing and fabricating semi-rigid bridge SMs was described in this paper. Digital impressions are made using an intraoral scanner, and it offers several advantages, including a reduction in potential discomfort or nausea associated with traditional elastic alginate impressions. Furthermore, intraoral scanning can be accomplished within a mere 2 min, significantly streamlining the overall operation time and it allows children to observe real-time imaging of their teeth on the display screen during the intraoral scanning procedure. Consequently, this enhances the overall comfort, engagement, interest and compliance of pediatric patients, effectively eliminating the apprehension they may have toward dental surgery [12]. A fully digital workflow was used to produce the SMs, reducing the extensive clinical and laboratory procedures and improving work efficiency. The CAD software was used in the 3D design of the SM, allowing for maximum precision with minimal potential for human error, which significantly increased the satisfaction of children and parents [13]. Another advantage of digital SMs is their suitability for electronic data storage and transmission. Identical copies of the original SM can be produced immediately to replace a fractured SM.

In this study, PEEK was used in the digitally cutting of the SM, eliminating multi-material interfaces, thereby potentially reducing the occurrence of SM fractures. PEEK has good mechanical properties, chemical stability, biocompatibility, and potential antibacterial properties [13, 14]. With its low modulus of elasticity of 4 GPa, PEEK offers a cushioning effect and reduce the stresses transferred to the abutment teeth [15]. Additional advantages of PEEK are the high wear resistance and fracture resistance [15]. Three-unit PEEK frameworks of the FDPs demonstrated deformation at 1200 N and fracture in the connector at 1383 N [16]. For healthy children aged 6–18, the maximum voluntary bite force in the anterior region was 140.0 N in girls and 169.0 N in boys [17]. Shiau et al. found that the mean values of unilateral bite force were 309.50 ± 193.75 N for boys and 219 ± 144.21 N for girls in ages from 7 to 20 years [18]. In this current study, spanning a 6-month follow-up period, revealed no instances of breakage or space loss with the semi-rigid bridge SMs, suggesting that PEEK was an appropriate material for SMs. It’s important to acknowledge that although PEEK’s opacity may limit its ability to achieve ideal aesthetics, it is better received by both parents and children when compared to metallic materials.

In the present study, a semi-fixed bridge SM was designed with a rigid connection end at the retainer’s full crown or band, based on the defect range of the abutment teeth, to ensure retention force. The non-rigid connection end was designed with a two-arm clasp to ensure stability and allow for arch and maxillary/mandibular development, making it suitable for children. The missing tooth area was designed according to the morphology of prematurely lost primary teeth, maintaining the mesiodistal dimension of the gap and restoring tooth morphology and masticatory function, which has advantages over traditional band loop SMs. Additionally, the pontic gingival types were designed with a 2–3 mm gap suspension to the gingiva, allowing for easy food passage and not affecting the normal eruption of permanent teeth. Follow-up appointments at T1, T2, and T3 showed no loose or broken SMs among the 15 participants, and there was no significant difference in the edentulous space. Overall, the children and their parents were satisfied with the SMs. One potential limitation of the semi-fixed bridge SM is that maintaining hygiene around the pontic can be challenging. During the T1 follow-up, one child exhibited poor oral hygiene with debris accumulation around the pontic gingiva. Therefore, it is necessary to provide parents and children with detailed oral hygiene guidance and emphasize the importance of flossing and interproximal brushing to prevent plaque accumulation, caries, and gingival inflammation.

## Conclusion

In this study, we fabricated 15 PEEK semi-fixed bridge SMs using CAD/CAM technology, which simplified the clinical steps and reduced the number of appointments required. PEEK material was more aesthetically pleasing compared to metal materials and was suitable for children with metal allergies. The semi-fixed bridge SM was effective in maintaining the space after the premature loss of primary teeth while allowing for the normal eruption of permanent successors. Our design offered the advantage of restoring masticatory function, which was not possible with traditional band loop SMs. However, long-term follow-up is still necessary to determine the effectiveness of this approach.

## Data Availability

The data that support the findings of this study are available from the corresponding author upon reasonable request.

## List of abbreviations

**CAD/CAM**: computer-aided design and manufacturing
**PEEK**: Polyetheretherketone
**SM**: space maintainer
